# Postoperative Pain Control After Cesarean Section by Continuous Infusion Pump System Versus Ropivacaine Hydrogel: A Prospective Randomized Clinical Trial

**DOI:** 10.3390/gels12030234

**Published:** 2026-03-12

**Authors:** Ji Eun Park, Hyen Chul Jo, Jong Chul Baek, Juseok Yang

**Affiliations:** 1 Department of Obstetrics and Gynecology, Gyeongsang National University Changwon Hospital, Changwon 51472, Republic of Korea; obgy@gnu.ac.kr (J.E.P.); cholida@naver.com (H.C.J.); gmfather@gmail.com (J.C.B.); 2 Department of Obstetrics and Gynecology, Gyeongsang National University School of Medicine, Jinju 52727, Republic of Korea; 3 Institute of Health Science, Gyeongsang National University, Jinju 52727, Republic of Korea

**Keywords:** thermosensitive hydrogel, poloxamer 407, polymer-based drug delivery, local anesthetic, cesarean section, postoperative analgesia

## Abstract

Objective: The objective of this study was to evaluate the efficacy of a ropivacaine-loaded poloxamer 407 (P407)-based thermosensitive hydrogel applied at the subfascial site compared with a continuous local anesthetic delivery system using a catheter for postoperative pain control after cesarean section (CS), in combination with standard intravenous patient-controlled analgesia (IV-PCA). Methods: This single-center, prospective randomized controlled trial included 72 pregnant women undergoing CS between April and October 2025. Participants were randomly assigned to receive either ropivacaine hydrogel or catheter-based ropivacaine infusion, both in conjunction with IV-PCA. Primary outcomes included numeric rating scale (NRS) pain scores at 3, 6, 12, 24, and 48 h postoperatively. Secondary outcomes included the time to first NSAID request and the cumulative use of rescue NSAIDs. Results: There were no significant differences in baseline characteristics between the groups. NRS pain scores did not differ significantly at any time point, although they varied significantly over time within each group. The hydrogel group showed a statistically significant delay in the time to first NSAID request (6.3 ± 5.1 h vs. 5.0 ± 6.1 h, *p* = 0.049) and higher cumulative NSAID use (2.4 ± 1.7 vs. 1.6 ± 1.2, *p* = 0.035). No serious complications were observed in either group. Conclusion: The ropivacaine hydrogel provided postoperative pain control comparable to that of the continuous catheter system, with no statistically significant differences in NRS scores observed between groups. Given its ease of use, absence of catheter-related concerns, and substantially lower total anesthetic dose, the P407-based hydrogel may represent a practical and patient-friendly alternative for post-cesarean analgesia.

## 1. Introduction

Cesarean section (CS) is the most frequently performed major surgery worldwide [1]. In the Republic of Korea, approximately 40% of deliveries among nulliparous women are conducted via cesarean section [2]. While the indications for CS can be categorized into maternal, fetal, and feto-maternal causes, autonomous decisions regarding the mode of delivery—so-called cesarean delivery by maternal request—have become increasingly common in recent decades [2]. Given its widespread implementation in modern obstetric practice, it is essential that every component of CS, including postoperative pain control, be grounded in evidence-based protocols [3,4].

Optimal control of postoperative pain has been shown to confer multiple benefits in recovery across various surgical procedures [5,6,7]. In addition to intravenous patient-controlled analgesia (IV-PCA) and rescue analgesics, continuous local anesthetic delivery systems have been widely adopted for pain management following CS. The On-Q PainBuster system (B. Braun), for instance, has been shown to significantly reduce opioid consumption, postoperative pain scores, and hospital stay durations [8,9].

These continuous local anesthetic systems deliver analgesics via a catheter inserted at the surgical site, typically at a preset flow rate, thereby achieving regional pain control. However, catheter-related complications and inconveniences remain critical limitations. Drug leakage around the wound or catheter, as well as the risks of surgical site adhesion, infection, or accidental catheter dislodgement, present notable drawbacks. Moreover, the need for catheter placement and subsequent removal is time-consuming and imposes added burden on both patients and medical staff.

An alternative method for achieving sustained regional anesthesia without catheter use involves the application of a thermosensitive hydrogel composed of poloxamer 407 (Kolliphor P407, BASF). When this FDA-approved polymer is mixed with local anesthetics such as ropivacaine and applied directly to the surgical site, it transitions to a gel at body temperature, enabling the slow release of the drug and prolonged analgesic effect. While several studies have reported non-inferior outcomes in postoperative pain management using this hydrogel system [10,11,12], no reports to date have evaluated the use of a P407-based ropivacaine hydrogel (Welpass^®^, Genewel Co., Ltd., Seongnam, Republic of Korea) specifically in cesarean deliveries.

Therefore, in this study, we aimed to evaluate the efficacy of hydrogel-based ropivacaine application compared to a catheter-based continuous local anesthetic delivery system for postoperative pain control following cesarean section.

## 2. Results and Discussion

This prospective, randomized clinical trial enrolled a total of 72 participants between April 2025 and October 2025. Participants were equally allocated to the study group and the control group, with 36 patients in each arm.

### 2.1. Baseline Characteristics

Table 1 presents the baseline demographic and obstetric characteristics of the participants. There were no statistically significant differences between the study and control groups in terms of maternal age, height, weight, body mass index (BMI), or gestational age at delivery. A total of 19 twin pregnancies were included (10 in the study group, 9 in the control group), with the remainder being singleton pregnancies (26 vs. 27, respectively).

The groups were well balanced regarding the history of previous cesarean section, including both primary and repeat CS. The mode of conception, whether natural or assisted (IVF or intrauterine insemination), did not differ significantly between groups.

Maternal complications such as gestational diabetes mellitus (GDM), hypertensive disorders (chronic hypertension, gestational hypertension, preeclampsia), thyroid dysfunction, threatened preterm labor requiring admission, and gestational thrombocytopenia were comparably distributed between the groups, with no statistically significant differences noted. These findings suggest effective stratified randomization, resulting in well-matched baseline characteristics. The distribution of operating surgeons was also comparable between groups (25/11 vs. 22/14, *p* = 0.46).

### 2.2. Primary Outcome: Postoperative Pain Scores

Pain intensity, as measured by the numeric rating scale (NRS), was the primary endpoint for assessing the efficacy of continuous postoperative analgesia. NRS pain scores were collected at 3, 6, 12, 24, and 48 h after surgery by trained ward nurses (Table 2). The highest pain scores were observed at 3 h postoperatively in both groups, with a steady decline over time. Although the control group showed slightly higher NRS at 3 h and the study group showed slightly higher values at 12 and 24 h, none of these differences were statistically significant (*p* > 0.05). At 48 h, pain scores were nearly equivalent between the groups. Between-group effect sizes were small at all postoperative time points (Cohen’s d range: −0.30 to 0.33), further supporting the absence of clinically meaningful differences in pain intensity.

**Figure 1:**
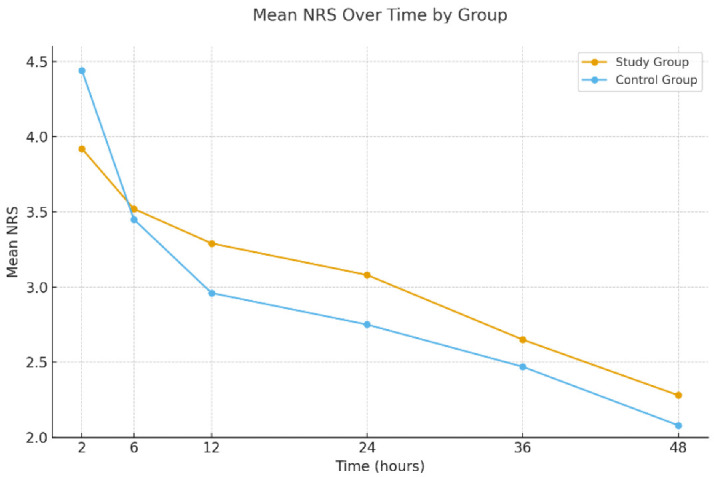
Mean numeric rating scale (NRS) scores for postoperative pain over time in the study and control groups. While both groups demonstrated a significant reduction in NRS scores over time, there was no statistically significant difference between the groups or in the interaction between time and group (repeated measures ANOVA, *p* = 0.965 and *p* = 0.103, respectively).

A repeated measure analysis of variance (ANOVA) was conducted to assess overall trends in pain over time (Figure 1). While no significant between-group difference was found (*p* = 0.965), there was a statistically significant within-group change in NRS scores across time points (*p* = 0.007), with a moderate effect size (η^2^ = 0.199). The interaction between time and group assignment was not statistically significant (*p* = 0.103), indicating that the trend of decreasing pain over time was consistent across both interventions.

### 2.3. Secondary Outcome: Use of Rescue Analgesics

Secondary outcomes included the cumulative use of additional nonsteroidal anti-inflammatory drugs (NSAIDs) and the time elapsed before the first request for rescue analgesia. As summarized in Table 3, the study group showed a statistically significant longer interval before the first NSAID administration compared to the control group (6.3 ± 5.1 vs. 5.0 ± 6.1 h, *p* = 0.049), indicating a delayed need for supplemental pain relief in those receiving ropivacaine hydrogel.

Furthermore, the cumulative number of NSAID doses administered postoperatively was also significantly higher in the study group (2.4 ± 1.7 vs. 1.6 ± 1.2, *p* = 0.035). Although this result suggests more frequent use of rescue analgesia in the hydrogel group, post hoc review revealed that two participants in the study group required seven NSAID administrations, which may be considered outliers. Excluding these two cases, the difference in cumulative NSAID use between groups was no longer statistically significant (2.0 ± 1.3 vs. 1.6 ± 1.2, *p* = 0.116), suggesting that the observed difference may have been driven by a small subset of high-frequency users rather than reflecting a group-wide trend.

The interquartile range (IQR) for cumulative NSAID use in the study group was 2.0, with Q1 and Q3 values of 1.0 and 3.0, respectively. Statistical outliers can be identified using either the Z-score method or the IQR-based approach. According to the IQR method, the upper boundary is calculated as follows: Upper bound = Q3 + 1.5 × IQR. Therefore, values exceeding 6 were considered statistical outliers. Two participants who required seven doses of NSAIDs during hospitalization exceeded this threshold and were thus reasonably classified as statistical outliers.

### 2.4. Perioperative and Postoperative Outcomes

Table 4 summarizes intraoperative and postoperative clinical outcomes. The average hospital stay was comparable between the two groups (4.4 ± 0.8 vs. 4.2 ± 0.5 days, *p* = 0.198), and spinal anesthesia was administered in nearly all cases. Operation time, hemoglobin levels before CS, and postoperative hemoglobin levels on POD1 and POD3 were also similar between the study and control groups, showing no statistically significant differences.

Regarding postpartum hemorrhage management, Bakri balloon tamponade was used in 5 patients in the study group and 2 in the control group (*p* = 0.191). Uterine artery embolization was performed in 2 patients, both in the control group. One case of transfusion occurred in each group. No cases of surgical site infection, surgical site dehiscence, urinary retention, or readmission for any reason were reported in either group. Post-dural puncture headache (PDPH) occurred in one patient in study group. Given the absence of moderate-to-severe intervention-related adverse events, formal severity grading according to the Common Terminology Criteria for Adverse Events (CTCAE) was not applicable in this cohort.

#### 2.4.1. Discussion on Main Findings

The primary finding of this prospective randomized clinical trial is that the application of ropivacaine with a P407-based thermosensitive hydrogel at the subfascial site is noninferior to a continuous local anesthetic delivery system using a catheter when used alongside standard IV-PCA in CS. The intensity of postoperative pain, assessed using the NRS at multiple time points, showed no statistically significant difference between the two groups. Repeated measures analysis confirmed that while pain intensity decreased over time in both groups, there was no significant interaction between group and time. Although this study was not designed as a formal noninferiority trial with a predefined noninferiority margin, the absence of clinically meaningful differences in NRS scores and secondary outcomes suggests comparable analgesic efficacy between the two interventions.

Interestingly, when evaluating secondary outcomes, both the time elapsed before the first request for rescue analgesics and the cumulative number of NSAID doses were significantly different between the two groups. The hydrogel group showed a longer interval before requesting additional analgesia and a higher cumulative number of NSAID uses, both with *p* values below 0.05. However, post hoc examination revealed that two participants in the study group required seven NSAID administrations, which greatly influenced the overall mean. When these outliers were excluded from the analysis, the difference in NSAID use between the two groups was no longer statistically significant, suggesting that the apparent difference may not represent a consistent group-wide pattern. These findings raise intriguing possibilities regarding the temporal profile of analgesic effect between the two interventions.

The apparently paradoxical finding of a longer time to first NSAID request but higher cumulative NSAID use in the hydrogel group may be explained by differences in drug release kinetics between the two delivery systems. The P407-based thermosensitive hydrogel undergoes temperature-dependent gelation, which may allow for relatively greater local anesthetic availability in the early postoperative period, potentially delaying the initial need for rescue analgesia. In contrast, catheter-based infusion provides a constant delivery rate that may maintain a more sustained analgesic plateau over time. These differing temporal profiles could contribute to the observed pattern of delayed first rescue request but higher overall NSAID consumption in the hydrogel group.

Adequate pain control is one of the most important final steps in surgical procedures including CS. While continuous delivery of local anesthetics through a catheter inserted during surgery has gained widespread use worldwide, since it has been reported to decrease the use of opioids, pain scores, and hospital stay [13,14], it has innate pitfalls that limit its convenience. Catheter placement and removal require time and skill, and although uncommon, the possibility of catheter-related infection remains a major concern. The relatively large total dose of local anesthetic used, along with potential systemic interactions, also represents a drawback. Furthermore, the presence of an indwelling catheter itself can cause discomfort and restrict patient mobility while requiring additional effort for dressing changes and wound care by healthcare providers.

In this study, all participants received identical fentanyl-based IV-PCA and as-needed NSAID protocols, in addition to the allocated pain control method. The hydrogel-based ropivacaine application achieved pain control comparable to the catheter-based system while using a markedly lower total anesthetic dose (22.5 mg vs. 750 mg). This reduction potentially minimizes the risk of systemic toxicity. Moreover, omission of catheter placement and removal saves time and reduces discomfort for both patients and medical staff.

Assessment of systemic toxicity of ropivacaine ideally requires measurement of serum concentrations, particularly peak plasma levels (Cmax), which were not evaluated in this study. Our primary objective was to assess clinical analgesic efficacy rather than pharmacokinetic profiles. We acknowledge that total administered dose does not directly equate to systemic toxicity, especially in the setting of continuous low-rate infusion via catheter. Nevertheless, cumulative dose remains one of the relevant determinants of systemic exposure in the absence of therapeutic drug monitoring. In routine clinical practice, reduction in total local anesthetic burden—while maintaining comparable analgesic efficacy—may represent a meaningful pharmacological advantage. Although we cannot infer differences in peak plasma concentrations or definitive safety superiority from our data, the markedly lower total ropivacaine dose used in the hydrogel group may warrant further investigation in future studies incorporating pharmacokinetic assessment.

However, the higher NSAID use observed in the hydrogel group, even if influenced by a few outliers, suggests that the long-acting analgesic effect of the hydrogel may be less sustained than that of the continuous infusion system, warranting further study to optimize formulation and dosage for extended efficacy.

#### 2.4.2. Discussion on P407-Based Hydrogel

The hydrogel-based system uses P407, a biocompatible, non-ionic, thermosensitive polymer that transitions from liquid to gel at body temperature, enabling sustained local drug release [15,16]. The clinical relevance of P407-based hydrogel primarily lies in its reversible thermosensitive gelation and sustained drug-release properties. Poloxamer 407 exists as a low-viscosity liquid at lower temperatures, such as under refrigerated storage conditions, where the polymer chains remain in a monomeric dissolved state. Upon exposure to body temperature, dehydration of the polypropylene oxide (PPO) blocks enhances hydrophobic interactions, leading to the formation of micelles composed of a hydrophobic PPO core and a hydrophilic polyethylene oxide (PEO) shell. With further temperature elevation, micellar packing and entanglement occur, resulting in a semi-solid gel structure. Although the gelation threshold is concentration-dependent, detailed physicochemical characterization was beyond the scope of the present study.

From a pharmacological perspective, the micellar architecture is particularly important for sustained drug release. Drug release from P407 hydrogels occurs through a combination of diffusion, gradual gel erosion, and encapsulation-mediated release from the hydrophobic micellar core. Local anesthetics such as bupivacaine and ropivacaine are relatively lipophilic molecules, which facilitates their partitioning into the hydrophobic core and contributes to prolonged release kinetics. Ropivacaine, an amide-type local anesthetic, exhibits moderate lipophilicity—lower than bupivacaine but higher than lidocaine—making it pharmacologically compatible with sustained release via micellar encapsulation.

In contrast, catheter-based infusion systems provide direct and continuous drug delivery at a fixed flow rate, resulting in relatively stable local concentrations but lacking the diffusion-controlled release profile seen in thermosensitive hydrogels. These differences in delivery kinetics may partly explain the temporal analgesic pattern observed in our study, in which the hydrogel group demonstrated a delayed request for rescue NSAIDs, suggesting effective early-phase drug availability. However, whether the sustained-release properties translate into superior long-term analgesia beyond the early postoperative period requires further investigation. A detailed pharmacokinetic comparison between hydrogel-based diffusion-mediated release and catheter-based infusion kinetics would be of significant interest but extends beyond the scope of the current clinical study.

#### 2.4.3. Discussion on Study Designs; Sample Size Calculation and Primary Endpoint

The authors adopted the study by Cusack et al., conducted in open gynecologic surgery, in which postoperative opioid consumption was the primary outcome, to estimate the expected effect size. In contrast, our trial predefined patient-reported pain intensity measured by the NRS as the primary endpoint. We acknowledge that basing the sample size calculation on a different clinical endpoint may raise concerns regarding methodological precision. However, several important considerations support this approach. First, to date, no randomized controlled trial has directly compared catheter-based continuous local anesthetic infusion with long-acting P407-based hydrogel analgesia in cesarean section patients. Therefore, we referred to the most clinically comparable surgical population available. Open gynecologic surgery shares substantial similarities with cesarean section in terms of abdominal wall incision, fascial manipulation, and the anatomical site of regional analgesic application, making it a reasonable surrogate model for estimation purposes.

Most importantly, as discussed by Nakagawa and Cuthill in 2007 [17], Cohen’s d represents a standardized, unitless measure of effect size that is independent of the measurement scale. Thus, although opioid consumption and NRS scores reflect distinct clinical endpoints, the standardized mean difference derived from prior data can serve as a pragmatic approximation when direct data are unavailable. The effect size range (d = 0.3–0.5) used in our calculation corresponded to a small-to-moderate effect and was intentionally selected as a conservative estimate to avoid overestimating treatment benefit. We acknowledge that the use of a surrogate effect size may introduce certain statistical limitations. Nevertheless, in the absence of cesarean-specific comparative data, this approach was considered methodologically reasonable and clinically justifiable at the study design stage.

NRS was set as the primary endpoint to assess pain intensity between groups in this study. Although the NRS does not capture qualitative characteristics of pain (e.g., burning, cramping, neuropathic features), its validity and reliability in quantifying overall pain intensity have been well established across diverse clinical settings. In particular, the NRS has been shown to be highly sensitive and stable compared with other commonly used pain measures. Mark P. et al. reported that the NRS demonstrated superior responsiveness and consistency in chronic pain populations compared to alternative pain scales [18]. Importantly, in the context of postoperative pain management after elective cesarean section, the NRS has also been validated for its clinical utility and reproducibility [19]. Furthermore, major perioperative analgesia studies and systematic reviews—including Berghella’s meta-analysis on perioperative gabapentin use for post-cesarean pain control—have adopted NRS pain scores as a primary outcome measure [20].

Given that our study aimed to compare overall analgesic efficacy between two regional analgesic techniques in a pragmatic clinical setting, the NRS was considered an appropriate and clinically meaningful primary endpoint. While it does not characterize pain quality, it provides a standardized, patient-centered measure of perceived pain intensity that allows for a comparison with existing literature and facilitates interpretation within current obstetric analgesia research.

#### 2.4.4. Discussion on Limitations and Strengths of This Study

This study has several limitations. First, blinding was not feasible because of the nature of the interventions, which might introduce bias. This study has several limitations. First, blinding was not feasible due to the inherent nature of the interventions, which may have introduced performance and detection bias. At the conceptualization stage of this trial, we deliberately decided not to implement blinding for two primary reasons: patient safety considerations and the potential for reciprocal bias introduced by artificial masking.

Because this study compared postoperative pain intensity between patients receiving hydrogel application and those managed with a catheter-based continuous infusion system, achieving blinding would have required the placement of a sham catheter in the hydrogel group. Although a saline-filled catheter system might have served as a placebo control, its insertion would have exposed participants to unnecessary procedural risks, discomfort, and potential catheter-related complications. Such an approach would have contradicted the ethical principle of minimizing harm and would have undermined the safety profile of this study.

Importantly, catheter-related discomfort and inconvenience constituted part of the clinical rationale for conducting this trial. Introducing a sham catheter solely for blinding purposes would have obscured a meaningful difference between the two interventions and potentially biased the evaluation of patient experience. Therefore, we considered that an unblinded design, while not ideal, was ethically and clinically more appropriate than implementing a sham procedure that might have caused additional harm.

Second, we did not assess the total fentanyl dose delivered via PCA, which could have strengthened the evaluation of overall analgesic effectiveness. Similar to the limitation regarding blinding, we were not able to assess the total fentanyl dose delivered or the number of bolus activations via IV-PCA after cesarean section. The elastomeric single-use PCA device used for all participants in this study did not have an automatic logging function to record bolus activations or cumulative delivered dose. In addition, residual volume of the solution was not measured in a standardized manner at the time of device removal. In routine clinical practice at our institution, the PCA device is typically removed on postoperative day 2, and in most cesarean section patients the reservoir is empirically nearly empty at the time of removal. In rare cases, early removal may occur due to opioid-related adverse effects such as severe nausea; however, no such premature removals were observed among the participants in this study.

We acknowledge that the absence of detailed adjuvant analgesic consumption data limits a more comprehensive evaluation of overall analgesic efficacy. Nevertheless, because the identical PCA protocol and device were applied uniformly to both groups, and no differential early discontinuations occurred, this limitation is unlikely to have substantially affected the primary comparative findings of our study.

Third, the concurrent use of IV-PCA and rescue NSAIDs might have masked subtle differences in perceived pain between groups.

Nevertheless, this study has strengths: it included both singleton and twin pregnancies, stratified by previous CS history to reflect clinical reality; surgical techniques were standardized to minimize inter-operator variability; and randomization with stratification ensured balance between groups.

## 3. Conclusions

In conclusion, our randomized clinical trial demonstrated that a single subfascial application of ropivacaine combined with a P407-based thermosensitive hydrogel provides postoperative pain control after cesarean section that is comparable to that achieved with a catheter-based continuous local anesthetic delivery system. No statistically significant differences in NRS pain scores were observed between groups. In addition, the hydrogel approach was associated with a substantially lower total anesthetic dose and eliminated catheter-related procedural burden. These findings suggest that ropivacaine hydrogel may represent a clinically practical and patient-friendly alternative to traditional continuous infusion systems in cesarean delivery.

Future studies with larger sample sizes and a predefined noninferiority framework, as well as evaluation of long-term analgesic duration and opioid-sparing effects, are warranted to further clarify its clinical role.

## 4. Materials and Methods

### 4.1. Study Design and Ethical Statements

This study was designed as a single-center, prospective, randomized clinical trial. The study protocol was approved by the Institutional Review Board of Gyeongsang National University Changwon Hospital (GNUH; IRB No. 2024-11-006). All participants provided written informed consent after being fully informed of this study by the investigator. This study was registered with the Clinical Research Information Service (CRiS, http://cris.nih.go.kr (accessed on 3 March 2026), ID: KCT0010404)

### 4.2. Patient Selection

We enrolled patients who underwent elective cesarean section (CS) at our institution. Both singleton and twin pregnancies were included. The exclusion criteria were: gestational age at delivery < 34 weeks, emergent CS with cervical dilatation > 4 cm, severe preeclampsia, triplet pregnancies, maternal age < 19 years, known contraindications or hypersensitivity to ropivacaine or other amide-type local anesthetics, known hepatic and/or renal failure, current or prior opioid dependence or misuse, and inability or unsuitability for study participation as determined by the investigator (e.g., poor cooperation)

### 4.3. CS Procedures

All CS procedures were performed by two obstetricians (J.E.P. and J.Y.). Transverse skin incisions were used in all cases (Joel-Cohen or Pfannenstiel), along with low-segment transverse uterine incisions. Fascia was dissected sharply (J.E.P.) or bluntly (J.Y.), and the peritoneum was opened sharply. Bladder dissection was not routinely performed. Uterine incision was widened laterally with two fingers (J.E.P.) or vertically by cranio-caudal traction (J.Y.). The uterus was closed with a single-layer Monocryl 1-0 suture, and the serosa was left unclosed. Peritoneum was closed, and fascia was closed using continuous Vicryl 1-0 (J.E.P.) or barbed Stratafix 1-0 suture (J.Y.). Skin was closed by subcuticular running suture using Nylon 3-0 (J.E.P., removed on postoperative day (POD) 3) or absorbable subcuticular skin stapler, INSORB (CooperSurgical) (J.E.P., J.Y.).

Spinal anesthesia was routinely used for all elective CS cases; when spinal access failed, general anesthesia was performed.

### 4.4. Randomization and Stratification

Participants were randomly assigned in a 1:1 ratio to one of two intervention groups: (1) P407 hydrogel-based ropivacaine (study group), or (2) catheter-based continuous local anesthetic delivery system (control group). Randomization was stratified based on history of prior CS to ensure balanced distribution of this key clinical variable across groups.

To implement this, a computer-generated randomization table was created in advance using a block randomization method with a fixed block size of four for each stratum (prior CS vs. no prior CS). The allocation sequence was prepared and maintained by an independent researcher not involved in patient recruitment or outcome assessment. Upon enrollment, participants were assigned to a group based on this pre-generated sequence. Enrollment began on 3 April 2025.

### 4.5. Sample Size Calculation

Based on a prior randomized clinical trial in gynecologic surgery [21], the effect size of postoperative opioid consumption was estimated with a Cohen’s d of 0.3 to 0.5. To achieve 80% power at an alpha level of 0.05 (one-sided hypothesis), 36 participants per group were required, yielding a total of 72 participants.

### 4.6. Intervention

In the study group, 6 g of P407-based thermosensitive hydrogel (Welpass^®^, Genewel Co., Ltd., Seongnam, Republic of Korea) was stored at room temperature in the operating room prior to use. During surgical preparation, while maintaining sterile conditions, the package was opened and 3 mL of 0.75% ropivacaine was directly injected into the prefilled 6 g hydrogel syringe.

The injection was performed using a lockable syringe system to prevent leakage during mixing. After injection, the two syringes were connected and the entire mixture was transferred back and forth between the syringes approximately 8–10 times to ensure homogeneous mixing. The mixing process was performed manually at a steady and controlled pace, equivalent to the standard movement of a 10 mL syringe, avoiding rapid or forceful agitation. This preparation process was completed during the surgical setup period before the start of the operation.

**Figure 2:**
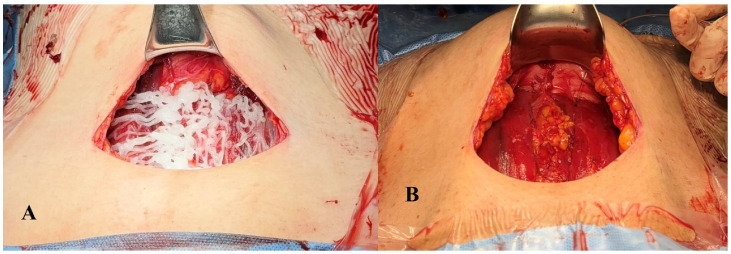
Subfascial application of two different local analgesic methods in cesarean section. (**A**) Ropivacaine-loaded thermosensitive hydrogel, consisting of 6 g of poloxamer 407 mixed with 3 mL of 0.75% ropivacaine (total 22.5 mg), was evenly spread across the subfascial surface before fascial closure. The hydrogel polymerized in situ, forming a stable gel matrix over the surgical field. (**B**) Continuous wound infiltration system with multi-orifice catheter was placed over the subfascial layer. The catheter was later connected to an elastomeric pump filled with ropivacaine for continuous delivery over 48 h.

After uterine closure and immediately prior to fascial closure, the mixed hydrogel was transferred back into the original P407 syringe. The syringe tip was replaced with a long, flexible applicator tip, and the hydrogel was evenly and broadly applied to the subfascial plane. Care was taken to distribute the material uniformly along the transverse fascial incision site. The application technique is demonstrated in the Supplementary Video S1 (Figure 2A).

In the control group, a continuous local anesthetic delivery system (MommyCaty Plus 100, MommyCaty, Seoul, Republic of Korea), a generic equivalent of the On-Q PainBuster, was used. After peritoneal closure, a catheter was inserted through the abdominal wall (left lateral and superior to the skin incision) using an introducer needle and placed under the fascia above the rectus muscle, the same location as the hydrogel application (Figure 2B; see also Supplementary Video S2). The catheter tip was advanced to approximately the depth corresponding to the second to third marking lines on the catheter shaft to ensure adequate distribution within the subfascial space. The catheter was secured at the skin entry site using 3-0 nylon sutures to prevent displacement. The elastomeric pump was filled with 100 mL of 0.75% ropivacaine (total dose: 750 mg) and set to deliver at a rate of 1 mL/h. The catheter was removed on POD 2.

All participants also received IV patient-controlled analgesia (IV-PCA) consisting of 600 mcg of fentanyl and antiemetics in 10 mL of normal saline, with a basal rate of 1 mL/h and a 1 mL bolus available every 15 min. In our hospital, we use elastomeric pump system of brand name AutoFuser (AceMedical, Gyeonggi-do, Republic of Korea). IV-PCA was initiated at the end of the CS and removed along with the IV line on POD 2. Additional analgesics—IV propacetamol 1 g or IM diclofenac 75 mg—were available on an as-needed basis.

Routine postoperative care was standardized in both groups, including sips of water immediately after surgery, Foley catheter removal on POD 1, and discharge on POD 3 if uneventful. Patients whose surgery started in the afternoon were admitted on the same day.

### 4.7. Outcomes

The primary endpoint was pain intensity assessed using NRS. Participants were instructed preoperatively on how to self-report pain using the NRS. Pain scores were collected at postoperative hours 3, 6, 12, 24, and 48 by trained ward nurses.

Secondary endpoints included the cumulative use of nonsteroidal anti-inflammatory drugs (NSAIDs) and the time interval from the end of surgery to the first NSAID request.

### 4.8. Data Collection

Demographic, obstetric, medical, and surgical history data were collected. Additional information, including gestational age at CS, indications for CS, and hospital stay duration, was extracted from medical records. Routine laboratory test results (POD 1 and POD 3) were also recorded.

### 4.9. Statistical Analyses

For categorical variables, we conducted χ^2^ test or Fisher’s exact test appropriately to compare the statistically significant differences between groups. Student’s *t* test or Wilcoxon’s rank sum test were employed for the continuous variables. A *p* value lower than 0.05 was considered to be statistically significant. Analyses was performed using R software version 4.5.1 (R Project for Statistical Computing, Vienna, Austria). All analyses were performed according to the intention-to-treat principle, including all randomized participants in their originally assigned groups.

## Figures and Tables

**Table 1:** Baseline maternal and perinatal characteristics of the study and control groups.

Characteristics	Study Group (*n* = 36)	Control Group (*n* = 36)	*p*-Value
Age (yr)	34.3 ± 3.8	35.1 ± 3.5	0.372
Height (cm)	162.7 ± 6.2	163.0 ± 5.8	0.799
Weight (kg)	75.9 ± 13.9	74.3 ± 12.8	0.640
BMI (kg/m^2^)	28.6 ± 4.2	27.9 ± 4.5	0.471
Week’s gestation at delivery	37.1± 1.3	37.2 ± 1.0	0.546
Singleton	26	27	1
Twin	10	9	
Repeat CS	20	16	1
First time CS	21	15	
Mode of conception			
Natural conception	18	24	0.216
In Vitro Fertilization	17	10	
Intrauterine insemination	1	2	
Maternal Complications			
Hypothyroidism	1	0	1
Subclinical Hypothyroidism	0	2	0.493
Hyperthyroidism	0	0	1
Subclinical Hyperthyroidism	1	0	1
GDM	4	3	1
T2DM	1	0	1
Chronic Hypertension	2	0	0.493
Gestational Hypertension	0	1	1
Preeclampsia	0	1	1
IIOC	1	0	1
Admission to maternity unit for the use of tocolytics	1	5	0.198
Gestational thrombocytopenia	0	1	1
Operating Surgeon			
J.Y.	25	22	0.46
J.E.P.	11	14	

BMI: Body Mass Index; CS: Cesarean Section; GDM: Gestational Diabetes Mellitus; T2DM: Type 2 Diabetes Mellitus; IIOC: Incompetent Internal Os of Cervix.

**Table 2:** Primary outcome: postoperative pain scores measured by numeric rating scale (NRS) at specified time points.

Postoperative Time (h)	NRS				*p*-Value
	Study Group (*n* = 36)	Control Group (*n* = 36)	Between Group Differences ^†^	Effect Size (Cohen’s d)	
3	3.9 ± 1.6	4.4 ± 1.7	−0.5 (−1.27, 0.27)	−0.30	0.163
6	3.5 ± 1.5	3.5 ± 1.6	0.0 (−0.66, 0.77)	0.00	0.648
12	3.3 ± 1.3	2.9 ± 1.1	0.4 (−0.23, 0.90)	0.33	0.267
24	2.9 ± 1.2	2.6 ± 1.1	0.3 (−0.18, 0.90)	0.26	0.212
48	2.3 ± 0.8	2.1 ± 0.8	0.2 (−0.23, 0.56)	0.25	0.381

^†^ Values are presented as differences (95% confidence intervals).

**Table 3:** Secondary outcome: use of rescue analgesics during the postoperative period.

NSAIDs Use	Study Group (*n* = 36)	Control Group (*n* = 36)	*p*-Value ^†^
Time elapsed for 1st NSAIDs request (h)	4.3 (3.0–8.0)	3.0 (1.8–6.0)	0.049
Cumulative uses of NSAIDs (*n*)	2 (1–3)	1 (1–2)	0.035

NSAID: Non-Steroidal Anti-Inflammatory Drug. Values are presented as median (interquartile range). ^†^ *p*-values were calculated using the Wilcoxon rank-sum test.

**Table 4:** Perioperative clinical outcomes.

Variables	Study Group (*n* = 36)	Control Group (*n* = 36)	*p*-Value
Hospital stay (day)	4.4 ± 0.8	4.2 ± 0.5	0.198
Anesthesia			1
Spinal	35	34	
General	1	2	
Postpartum hemorrhage			0.191
BAKRI balloon tamponade	5	2	
Uterine Arterial Embolization	0	2	
Hemoglobin before CS (g/dL)	11.2 ± 1.4	11.4 ± 1.4	0.682
Hemoglobin POD1	11.0 ± 1.6	10.8 ± 1.5	0.542
Hemoglobin POD3	10.5 ± 1.7	10.1 ± 1.7	0.317
Transfusion	1	1	1
Operation time (min)	36.1 ± 12.3	35.3 ± 11.0	0.864
Surgical site infection	0	0	n/a
Readmission for any reasons	0	0	n/a
Surgical site dehiscence	0	0	n/a
Urinary retention	0	0	
PDPH	1	0	1

CS: Cesarean Section; POD: Post-Operative Day; PDPH: Post-Dural Puncture Headache.

## Data Availability

The original contributions presented in this study are included in this article. Further inquiries can be directed to the corresponding author.

## Supplementary Materials

The following supporting information can be downloaded at: https://www.mdpi.com/article/10.3390/gels12030234/s1, Video S1: Application of the ropivacaine hydrogel to the surgical site during cesarean section; Video S2: Placement of the catheter for continuous local anesthetic administration.
